# ZD7288, a blocker of the HCN channel family, increases doubling time of mouse embryonic stem cells and modulates differentiation outcomes in a context-dependent manner

**DOI:** 10.1186/s40064-016-1678-7

**Published:** 2016-01-16

**Authors:** Anna Omelyanenko, Petra Sekyrova, Michael Andäng

**Affiliations:** Department of Physiology and Pharmacology, Karolinska Institutet, 171 77 Stockholm, Sweden; Central European Institute of Technology, Masaryk University, Kamenice 735/5, 625 00 Brno, Czech Republic

**Keywords:** Embryonic stem cells, Ion channel modulator, ZD7288, Differentiation, Proliferation, Pluripotency, Cell cycle, Serum

## Abstract

Pluripotent stem cells are the starting cell type of choice for the development of many cell-based regenerative therapies due to their rapid and unlimited proliferation and broad differentiation potential. The unique pluripotent cell cycle underlies both these properties. Hyperpolarization-activated cyclic nucleotide-gated cation (HCN) family channels have previously been reported to modulate mouse embryonic stem cell (ESC) proliferation and here we characterize the effects of HCN inhibitor ZD7288 on ESC proliferation and stem cell identity. The doubling time of cells treated with the HCN blocker increased by ~30 % due to longer G1 and S phases, resulting in a nearly twofold reduction in ESC numbers after 4 day serum-free culture. Slower progression through S phase was not accompanied by H2AX phosphorylation or cell stalling at transition points, although EdU incorporation in treated cells was reduced. Despite the drastic cell cycle perturbations, the pluripotent status of the cells was not compromised by treatment. Cultures treated with the HCN blocker in maintenance conditions maintained pluripotency marker expression on both RNA and protein level, although we observed a reversible effect on morphology and colony formation frequency. Addition of ZD7288 in differentiating media improved FBS-driven differentiation, but not directed differentiation to neuroectoderm, further indicating that altered cell cycle structure does not necessarily compromise pluripotency and drive ESCs to differentiation. The categorically different outcomes of ZD7288 use during differentiation indicate that cell culture context can be determinative for effects of ion-modulatory molecules and underscores the need for exploring their action in serum-free conditions demanded by potential clinical use.

**Electronic supplementary material:**

The online version of this article (doi:10.1186/s40064-016-1678-7) contains supplementary material, which is available to authorized users.

## Background

As we draw closer to fulfilling the promise of regenerative medicine, more and more cell therapy techniques are relying on a pluripotent cell source (Tabar and Studer 2014). Whether embryonic stem cells (ESCs) or induced pluripotent stem cells (iPSCs) are used, their capacity to generate any cell type in the adult body, together with their unlimited and robust proliferation, present both the main advantages and some of the greatest challenges to therapy development. These properties are harnessed and kept in check by culture in specific conditions, permissive and instructive of timely differentiation to the desired cell types. Clinical use of the resulting cells puts additional requirements on protocol development, such as the use of well-defined serum-free media components. Balancing the many biological and regulatory requirements on differentiation strategies makes understanding of the underlying stem cell biology and the interplay between stem cell properties and culture conditions essential to success in bringing forward new cell-based therapies.

The cell cycle of embryonic stem cells is characterized by a lack of a G0 and shortened G1 and G2 phases. This has major implications for the underlying regulatory network, as most conventional regulatory factors, which cycle during cell cycle progression in differentiated cell types do so to a significantly lower extent in embryonic stem cells. ESCs lack hypophosphorylated RB protein (Savatier et al. 1994), show constitutive CDK2, cyclin A and cyclin E activity (Stead et al. 2002), lower APC/C activity (Ballabeni et al. 2011), and constitutively express geminin (Yang et al. 2011). Altogether, these biochemical characteristics of ESCs lead to a shortened G1 and G2. Pluripotent cells also respond differently to damage pathway induction, by, for example, failing to upregulate p21 in response to DNA damage-induced p53 accumulation, which leads to downregulation of Nanog and differentiation, instead of G1/S cell cycle arrest (Hyka-Nouspikel et al. 2012; Lin et al. 2005).

There is a strong link between cycling and maintenance of stem cell identity, as differentiation is invariably accompanied by a lengthening of the cell cycle, and reprogramming, conversely, by its shortening (Li and Kirschner 2014). This variability in transit time is accounted for by lengthening of the gap phases, G1 and G2, while S and M phases are similar in length in pluripotent and differentiated cells (Stead et al. 2002; Chen et al. 2015b). G1 in particular has received a lot of attention, with early work showing that embryonal carcinoma cells are only responsive to retinoic acid differentiation while in G1 (Mummery et al. 1987). Since then, a shorter G1 was tied to self-renewal (Becker et al. 2006) and improved reprogramming capacity (Ruiz et al. 2011; Guo et al. 2014). The initial observation gained fresh backing with recent RNA sequencing studies showing that differentiation and lineage specification transcription factors are preferentially transcribed in G1 (Singh et al. 2013). Intriguingly, interfering with the cell cycle, most often through CDK2 inhibition, has showed varying effects on differentiation and self-renewal. While lengthening G1 with small molecule inhibition of CDK2 has resulted in impaired self-renewal in some studies (Koledova et al. 2010), other studies using different small molecule CDK2 inhibitors or overexpression of p21 and p27, native CDK2 inhibitors, failed to show similar effects (Li et al. 2012; Stead et al. 2002).

We and others (Andang et al. 2008a; Lau et al. 2011; Rodriguez-Gomez et al. 2012) have previously reported that modulation of ion fluxes in embryonic stem cells can induce alterations in proliferation kinetics with varying effects on stem cell fate. While knockout of GABAA beta3 subunit, which reduced ESC proliferation in vitro, results in viable mice (Liljelund et al. 2005), suggesting that stem cell potential is not compromised in the absence of GABA signalling, inhibition of T-type calcium channels slows down proliferation and induces premature ESC differentiation (Rodriguez-Gomez et al. 2012). In a 2011 study by Lau et al. (Lau et al. 2011) block of the hyperpolarization-activated cyclic nucleotide-gated cation channel (HCN) family was found to slow down mESC proliferation, however the impact this had on pluripotency maintenance was not investigated.

The HCN family of channels consists of four members with varying tissue expression patterns (Calejo et al. 2014) and gating and activation kinetics (Biel et al. 2009). HCN1, 2 and 4 have been extensively studied for their functions in rhythm generation and regulation of membrane polarization in neurons and the sinoatrial node, whereas the function of HCN3 is less clear (DiFrancesco and DiFrancesco 2015). HCN3, which is widely expressed over the entire organism at low levels, was the only HCN family member found to be expressed on a protein level in ESCs (Lau et al. 2011). The channel activates at voltages negative of resting membrane potential and initiates repolarization, with opening and closing kinetics varying depending on intracellular cAMP levels. In this work we looked more closely at the effect of ZD7288, a widely used tool compound for modulation of the HCN current, on the ESC cell cycle and characterized the previously uninvestigated effects of this compound on stem cell fate.

## Results

### ZD7288, an HCN channel blocker, reduces ES cell proliferation by increasing doubling time

**Fig. 1:**
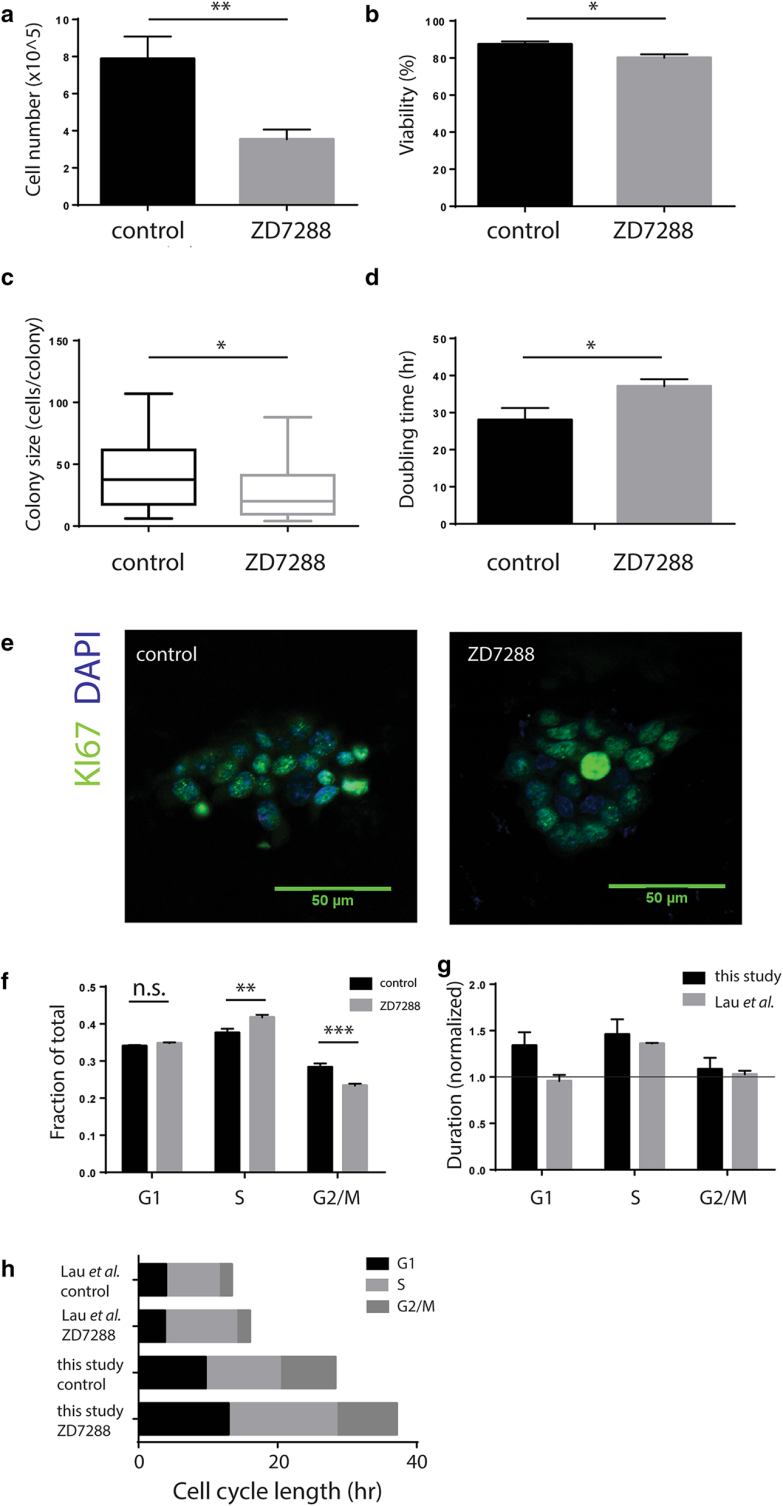
Blocker of HCN family channels, ZD7288, reduces ESC proliferation by lengthening the G1 and S phases of the cell cycle. Culture in the presence of 10 μM HCN inhibitor ZD7288 resulted in reduced mESC cell numbers after 4 days in suspension (Student’s t-test; p < 0.01; n = 9) (**a**) with a minimal reduction in viability (Student’s t-test; p < 0.05; n = 9) (**b**). Cells cultured adherently at subclonal densities formed smaller colonies (Mann Whitney test; p < 0.05; n = 3) (**c**). Despite a significantly longer doubling time (sum of squares f-test; p < 0.05; n = 9) (**d**) the cells did not exit the cell cycle, as indicated by ubiquitous KI-67 expression in both control and treated cultures (**e**). Flow cytometry for DNA content (**f**) showed an accumulation of cells in the S phase (Student’s t-test; p < 0.01; n = 3), and a relative reduction in the number of cells in G2/M (Student’s t-test; p < 0.01; n = 3). Doubling time and cell cycle fraction were used to calculate residence time in each cell cycle phase for control cultures and cultures treated with 10 μM ZD7288 (**g**). Comparison of cell cycle structure in our study and previous study by Lau et al. is summarized in (**h**)

When ES cells were cultured in the presence of an HCN blocker (ZD7288), cell proliferation was significantly reduced with a nearly twofold reduction in cell numbers after 4 days of culture (7.9 ± 1.2 × 10^5^ cells/ml control culture, 3.5 ± 0.5 × 10^5^ cells/ml treated culture, p < 0.01), and only a small (87.4 ± 1.4 % control vs 80.1 ± 1.8 % treated, p < 0.01) reduction in viability (Fig. 1a, b). Colony size was correspondingly reduced when cells were grown at subclonal densities, decreasing from an average of 44 to 27 cells per colony (43.8 ± 6.2 cells/colony in control, 27.0 ± 3.5 cells/colony in treated, p < 0.05) 5 days after plating (Fig. 1c). Doubling time, which was ~28 h for untreated bulk cells [in line with previously published findings for this culture system (Tamm et al. 2013)], was increased to ~37 h for ZD7288 treated cells (Fig. 1d).

Since the slight reduction in viability could not account for the drastically reduced cell count, we looked for possible alterations in cell cycle phase distribution due to ZD7288 treatment. The G0 phase is usually absent from the ESC cell cycle and positive KI-67 staining indicated that this is also the case for ZD7288 treated cells (Fig. 1e). To look at the phases of the active cell cycle we performed flow cytometry for DNA content. We observed a significant increase in S phase upon ZD7288 treatment (37.6 % in control, 41.8 % in treated, p < 0.01), which is in agreement with the findings of Lau et al. (2011), even though the two studies were carried out under different conditions. In contrast to Lau’s findings, where the fraction of cells in G1 was reduced and of those in G2/M unchanged, we saw that the increase in the S phase fraction was accompanied by a reduction in the G2/M compartment (28.3 % in control, 23.4 % in treated, p < 0.001) (Fig. 1f). Using the calculated doubling time and cell cycle fractions, we calculated the time spent in each of the phases. In our hands, both G1 and S phases of the cell cycle were extended due to ZD7288 treatment, whereas analysis of data from Lau et al. indicated an appreciable increase in the length of S phase only (Fig. 1g, h).

### DNA replication in S phase is altered in ZD7288 treated cells

**Fig. 2:**
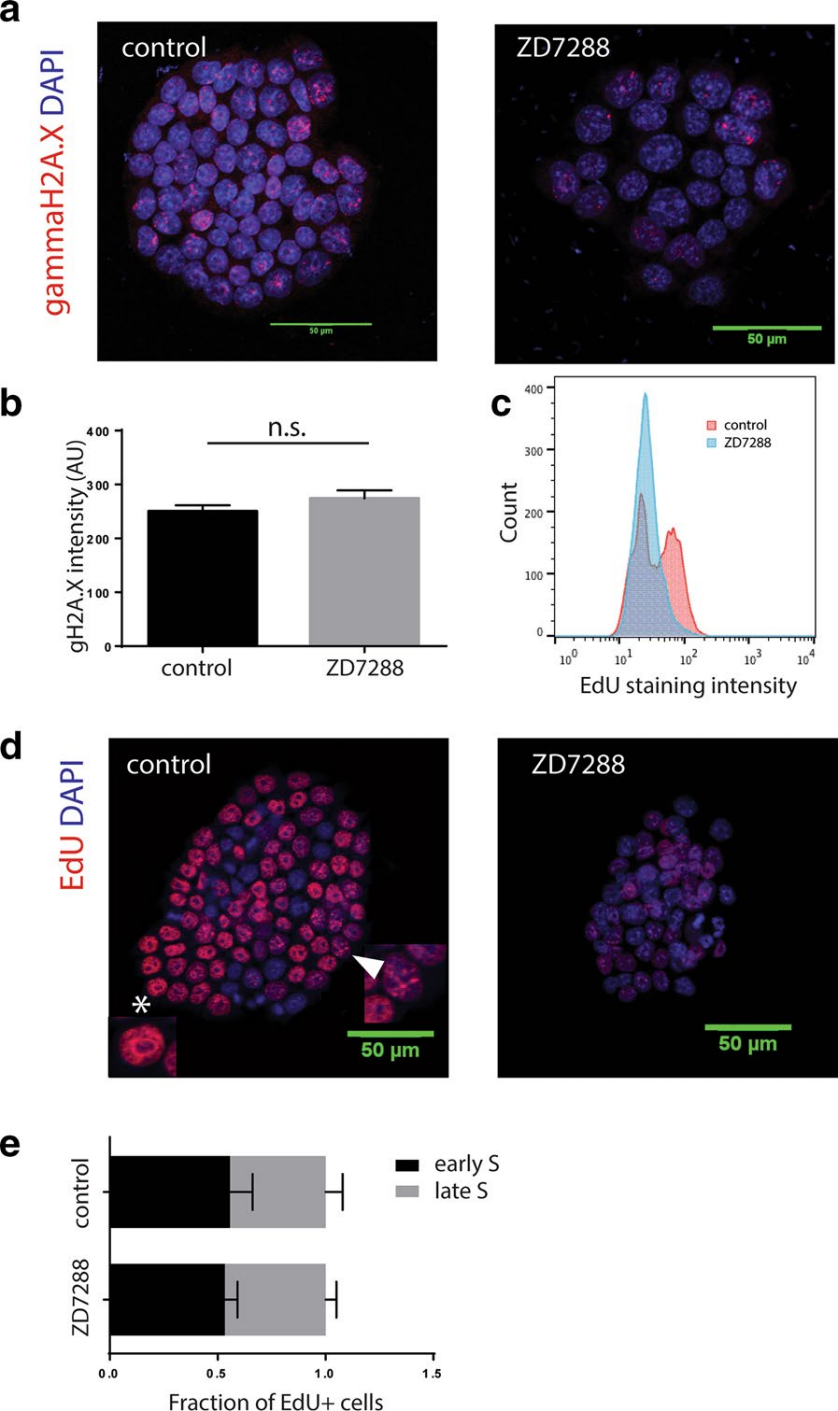
S-phase lengthening is not mediated by increased gammaH2AX or altered S-phase progression, but is accompanied by reduced nucleoside incorporation. Control cells and cells treated with ZD7288 for 5 days were stained for gammaH2AX (**a**), with no difference in staining intensity (Student’s t-test; not significant; n = 3) (**b**). Cells grown in bulk with and without the addition of ZD7288 were pulsed with 10 μM EdU prior to fixation and stained for flow cytometric analysis. Treated cells incorporated less EdU (p < 0.05, n = 3) (**c**). Cells grown adherently for 4 days in control or ZD7288 conditions were also pulsed and stained for EdU (**d**), confirming lower levels of incorporation. Cells with EdU incorporation pattern typical of early S-phase (**d**, *star* and *inset*) and mid-late S-phase (**d**, *arrowhead* and *inset*) in both conditions were counted (**e**) with similar distribution of cells between early and late S under both conditions (Student’s t-test; not significant; n = 3)

It has previously been shown that gamma-aminobutyric acid (GABA) can induce proliferative arrest in ESCs via a DNA-damage-independent induction of the DNA damage response (DDR) pathway and histone H2AX phosphorylation (Andang et al. 2008a). Since both ZD7288 and GABA are expected to influence ion fluxes, affecting membrane polarization dynamics, they may activate common downstream pathways to attenuate proliferation. To test this we performed flow cytometry and immunohistochemistry for γH2AX, but did not find increased histone phosphorylation levels in treated cells (Fig. 2a, b).

To examine the alterations in S-phase, cells were pulsed with EdU to allow observation of nucleotide incorporation during DNA replication. When we looked at nucleotide incorporation using flow cytometry, fewer cells in the blocked culture were deemed EdU positive (37.3 ± 2.4 % in control, 19.7 ± 3.6 % in treated, p < 0.05), despite a larger fraction of cells being in the S-phase based on DNA content (see Fig. 1f). This was due to much lower EdU incorporation levels in the HCN-blocked cells resulting in a completely altered EdU incorporation profile. Whereas control cells showed two clear peaks corresponding to EdU incorporating and non-incorporating cells, all ZD7288 treated cells fell within a single wide peak (Fig. 2c, d). The observed change in EdU incorporation profile could be due to stalling at a particular point within S-phase, slower DNA replication, or reduced EdU availability.

To investigate potential accumulation of cells at a particular point in S-phase, we looked at the distribution of cells between early and late S phase using a previously described method where cells are staged based on EdU incorporation pattern (Solovei et al. 2006). The sub-S phase distribution was not affected, with 55.7 ± 10.5 % of cells in early S in the control condition and 53.3 ± 6. 0 % in early S in ZD7288 treated cultures (Fig. 2d, e). This indicated that in the presence of the HCN inhibitor, cells continue replicating DNA and progressing through S-phase, while reduced EdU incorporation suggests that this process occurs at a slower rate than in control conditions, supporting our findings of a longer S-phase.

### Treatment with ZD7288 decreased colony formation frequency but did not alter expression of the core pluripotency genes

**Fig. 3:**
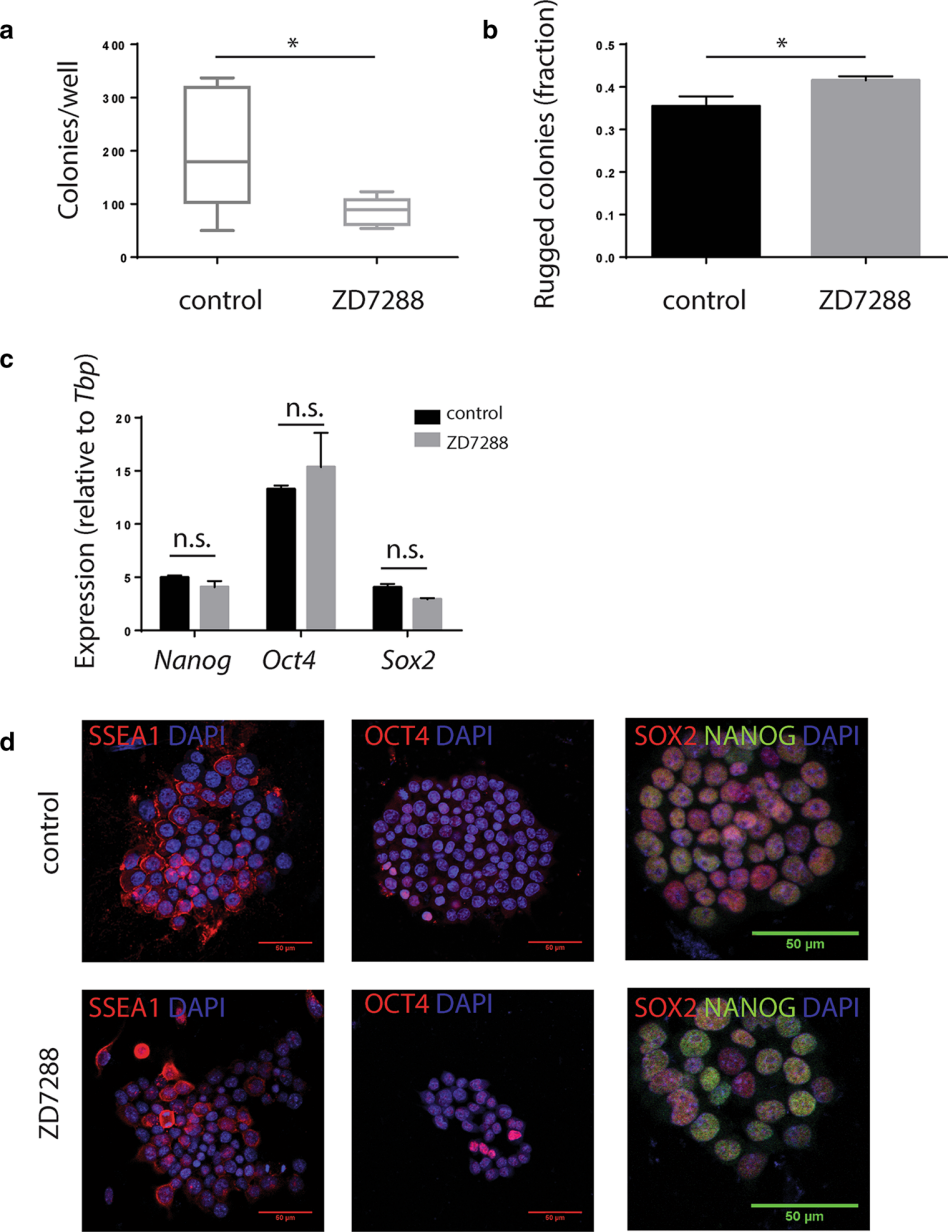
HCN blocker treatment reduced colony formation frequency but did not induce differentiation. mESC plated at subclonal density were allowed to form colonies in control or ZD7288-containing media for 5 days and number of colonies per well was counted (Mann Whitney test; p < 0.05; n = 6) (**a**). Morphology was scored and fraction of rugged colonies calculated (Student’s t-test; p < 0.05; n = 6) (**b**). At the same time point, RNA was collected for qPCR analysis of pluripotency marker expression, which was unchanged (Student’s t-test; not significant; n = 3) (**c**). Finally, fixed cell were stained for pluripotency marker expression, with cells in both culture conditions showing expression (**d**)

To examine effects of HCN inhibitor treatment and subsequent cell cycle lengthening on ESC self-renewal in maintenance media, ESCs were plated at subclonal density and allowed to form colonies for 4 days in the presence or absence of ZD7288. Although ALP expression was not altered, colony numbers were reduced upon treatment (Fig. 3a), and fraction of ALP positive colonies with rugged edges increased (Fig. 3b). To investigate if these effects on morphology were accompanied by loss of pluripotency, we looked at expression of pluripotency markers by qPCR and immunohistochemistry. qPCR analysis showed no significant differences in pluripotency marker expression between treated and untreated cells (Fig. 3c), suggesting that altered colony morphology was not due to differentiation. This was confirmed on the protein level, with colonies staining positive for SSEA1, OCT4, SOX2 and NANOG (Fig. 3d) in both culture conditions. To further indicate that the self-renewal potential of the ESCs was not compromised, the relative ratio of rugged and smooth colonies was restored when ZD7288 treated cells were replated and allowed to form colonies in ZD7288-free media (data not shown).

### HCN inhibitor ZD7288 affects ES cell differentiation outcomes in a context-dependent manner

In addition to self-renewal, the ability to differentiate into the 3 germ layers is a critical property of ESCs. To investigate how this property was affected by treatment, we looked at differentiation potential under unbiased conditions and during directed differentiation with and without ZD7288.

**Fig. 4:**
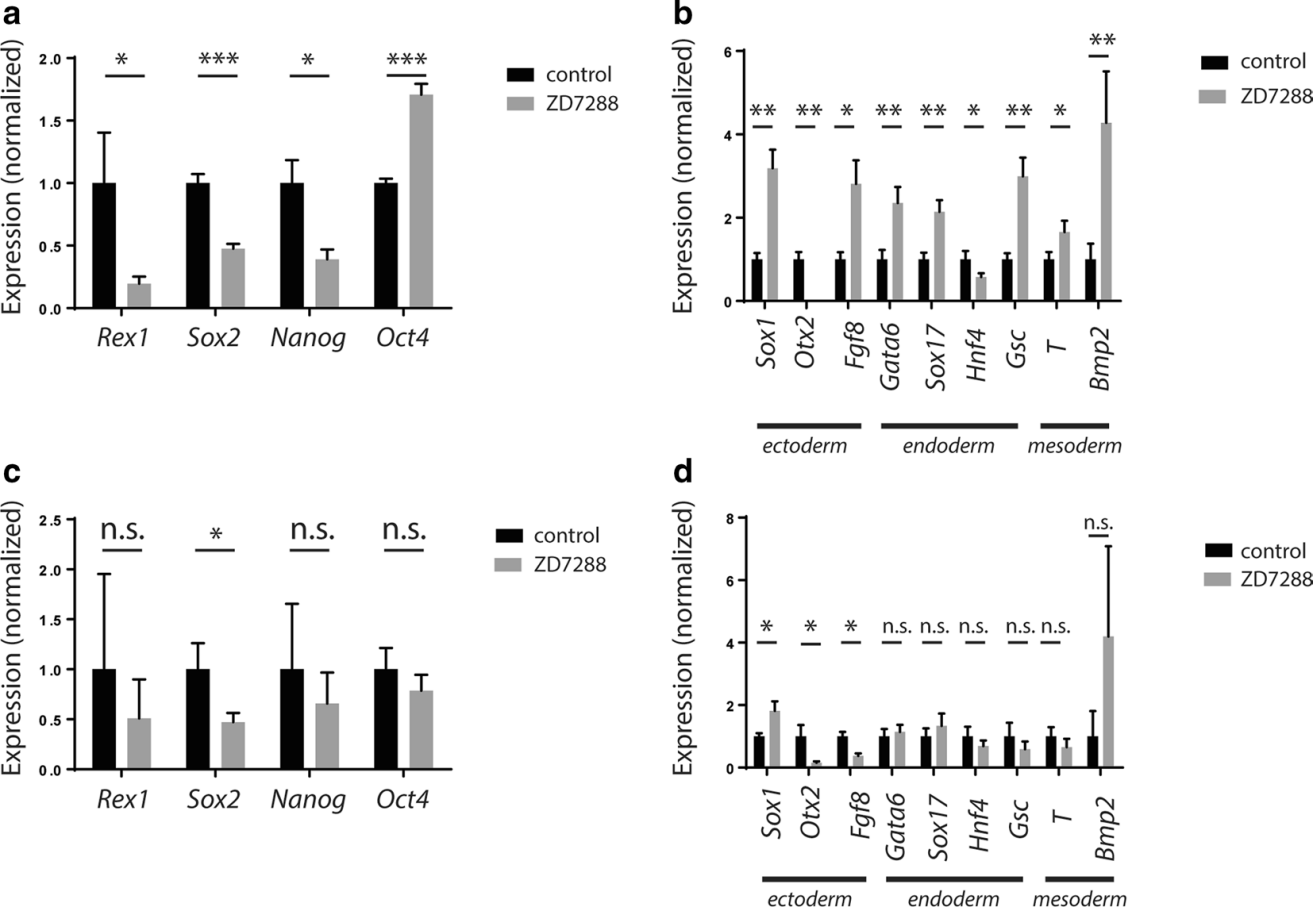
Administration of ZD7288 facilitated differentiation in a protocol-dependent manner. Relative expression levels of pluripotency genes (**a**) and a panel of differentiation genes (**b**) in cells spontaneously differentiated using FBS. Relative expression levels of pluripotency genes (**c**) and differentiation genes (**d**) using neurodifferentiation protocol. Ct values were normalized to *Tbp* expression. Student’s t-test; * p < 0.05; ** p < 0.01; *** p < 0.001, n.s. not significant; n = 3

To test differentiation under unbiased conditions, ESCs were plated on laminin in FBS containing media without LIF supplementation in the presence or absence of ZD7288. qPCR for differentiation and pluripotency genes 5 days following induction of FBS differentiation showed that ZD7288 treated cells upregulated lineage genes specific for the 3 germ layers and extraembryonic endoderm (Fig. 4b) to a significantly higher degree than cells differentiated in the absence of the compound. Concomitantly, pluripotency-specific genes were downregulated to a significantly greater extent in treated culture, with the exception of *Oct4*, which was expressed at higher levels (Fig. 4a), consistent with extraembryonic endoderm induction (Wang et al. 2012).

Surprisingly, when directed differentiation towards the neural lineage was induced by culturing cells in LIF free media supplemented with B27, we did not see improved differentiation under ZD7288 treatment conditions, as was observed with unbiased differentiation. There were no significant differences in expression of most pluripotency markers (Fig. 4c) between control and ZD7288-treated conditions, and no significant increase in expression of most differentiation markers (Fig. 4d) in the HCN block condition. In the case of neural markers, of the 3 markers tested two, *Otx2* and *Fgf8*, were expressed at significantly lower levels in the treated cells than control, and the only significantly downregulated pluripotency marker was *Sox2*, which was expected to be high in neural cells. In direct opposition to what was observed under FBS differentiation, the use of the HCN inhibitor during differentiation did not improve the extent of neural differentiation. These data may indicate altered differentiation capacity in response to ZD7288 treatment, or the emergence of subpopulations differentially responsive to the compound.

## Discussion

We have shown that treatment of mouse embryonic stem cells with the HCN channel family inhibitor ZD7288 results in longer cell cycle transit time. Analysis of cell cycle phase distribution showed that the relative number of cells in the G2/M phase declined upon treatment, while the S phase fraction increased, indicating a relative lengthening of the G1 and S phases with no change in G2/M due to treatment.

The HCN blocker-induced increase in S-phase residence time was not due to accumulation of cells in either early or late S, arguing against the requirement for HCN activity for S to G2/M progression proposed by Lau *et al.* as the underlying cause of the observed S-phase accumulation. Our experiments showing decreased EdU incorporation rather suggest a slowdown in incorporation of new nucleosides, whether due to slowed replication or impaired import, resulting in slower progression through the entire S-phase. The fact that both S and G1 phases were lengthened, and to a similar degree, argues that HCN blocker affects events critical in both phases. Two intriguing possibilities are nutrient import and chromatin folding. The 3D structure of the DNA and its organization on scaffolding proteins has important implications for allowing or impeding access of transcriptional and replication machinery to the DNA, and has been shown to be sensitive to its ionic environment, especially with regard to mono- and divalent cation concentrations (Strick et al. 2001). Nutrient import, on the other hand, is known to occur via sodium-coupled co- and symporters for a number of critical molecules, such as glucose (Chen et al. 2015a), nucleosides (Choi and Berdis 2012), and amino acids (Bror 2014). HCN channels carry cation currents and are instrumental in regulating sodium fluxes during action potentials in electrically active cells (Biel et al. 2009), and could thus play a role in sodium homeostasis in ESCs, influencing one or both of the above. Interestingly, ZD7288 has also been shown to inhibit the voltage-activated Na_v_1.4 sodium channels (Wu et al. 2012), although these channels have not been detected in ESCs (Wang et al. 2005).

Regardless of the underlying cause, the observed significant slowdown in cell cycle progression can be expected to compromise self-renewal. However, we did not observe this in our study, with both treated and untreated cells expressing the core pluripotency markers to similar degree. In fact, significant variation in doubling time of multiple ESC lines stemming from variation in culture conditions has previously been reported, with cell cycle transit times varying from as short as 13 h to as long as 34 h (Tamm et al. 2013). Despite the difference, ESCs grown in all culture conditions have been shown to retain their stem cell properties and could contribute to chimeras (Andang et al. 2008b; Ying et al. 2008; Smith 1991), indicating that a prolonged doubling time does not lead to loss of stem cell potential per se, and supporting our findings that pluripotency marker expression is unchanged when doubling time is increased as a response to treatment.

Interestingly, our data show that spontaneous differentiation using serum, even to ectodermal lineages, but not directed differentiation to neuroectoderm in defined medium, was improved when ZD7288 was added to culture media. The ability of ZD7288-treated cell to upregulate markers from all lineages in response to FBS differentiation indicates that the differentiation potential of the cells is not skewed by the treatment, further supporting our finding that stemness properties are not perturbed by the HCN blocker. The observed difference in the functional outcomes of ZD7288 treatment under the two differentiation conditions could be due to a number of different factors. It is possible that the differentiated progeny responds differently to HCN block in the two conditions, and has a growth advantage over undifferentiated ESCs in FBS but not in neural differentiation, resulting in higher degree of differentiation in the FBS culture treated with ZD7288.

A more intriguing possibility to consider is the interaction of serum and ion modulation in regulating the cell cycle and thereby differentiation propensity. Whereas we observed a G1 lengthening as a response to treatment in maintenance conditions, this was not reported in previously published work. One key difference between our study and the earlier study by Lau et al. are media components, with us using serum-free media in contrasts to FBS supplemented media used previously. Serum is a potent modulator of the length of the cell cycle, and the G1 in particular. When looking at cell cycle profiles and doubling times of cells grown in serum-free conditions, such as in our experiments here, and previously published data for FBS supplemented conditions (Savatier et al. 2002; Jovic et al. 2013), the calculated S-phase lengths are similar at around 11 h (70 % of 16 h with FBS, 38 % of 28 h in serum-free) while the G1 is extended nearly threefold to 9 h in the absence of FBS (20 % of 16 h with FBS, 35 % of 28 h in serum-free). The longer G1 in serum-free cultured ESCs could impose drastically different requirements for HCN function than the short G1 in FBS cultured cell, especially if the channel family plays a role in regulating nutrient transport and biomass growth. In such a case, cells under HCN block face an entirely different cell cycle landscape depending on the presence or absence of serum in culture medium.

It has previously been discussed that the relative ratio of G1:S length may be critical for pluripotency maintenance, with a lower ratio being linked to improved self-renewal (Hindley and Philpott 2013). For cells induced to differentiate in serum-free conditions under HCN block, this ratio is only slightly lowered, and they would thus be expected to differentiate with dynamics similar to untreated cells, consistent with our observations. FBS differentiated cells treated with the HCN blocker would have a drastically lowered G1:S ratio, which should result in improved self-renewal. However, if HCN function is critical in G1, under HCN blockade the absence of a G1/S checkpoint in mESCs could result in precocious S-phase entry accompanied by stress and induction of differentiation. In budding yeast, precocious S-phase entry induces S-phase lengthening and Rad53 induction, which is required for viability maintenance (Sidorova and Breeden 2002). CHK2, the mammalian homologue of Rad53, is required for histone H2AX phosphorylation in mitosis (Tu et al. 2013). This is of particular interest since H2AX phosphorylation is necessary for S-phase accumulation of ESCs in response to GABA modulation as previously described (Andang et al. 2008a).

In future experiments it would be of great interest to rigorously test the potential role of HCNs in nutrient transport in ESCs maintained in FBS supplemented media. This work supports the idea that G1 lengthening, in and of itself, does not compromise the pluripotent state, and other factors, such as nutrients and permissive growth factor signalling, provide an essential layer of fate control. Cell cycle effects are an important consideration when designing cell culture protocols for stem cell expansion and differentiation, and significant attempts are being made to incorporate them into our understanding of the path from pluripotency to commitment (Li and Kirschner 2014). Mapping the cell fate landscape and finding stable states will facilitate controlled, scalable and reproducible generation of desired cell types using defined, serum-free culture conditions, a necessity as cell-based therapies move into the clinic. However, as we focus on the signalling pathways that direct the switch between desired states, we should not forget the basic cell physiological requirements that need to be fulfilled to make the transitions.

## Conclusion

In this work we show that treatment of mouse ESCs with the HCN-channel family blocker ZD7288 induces cell-cycle lengthening, with an extended G1 and S phases. Interestingly, neither the altered cell cycle kinetics nor ionic perturbation compromised ESC self-renewal, as cells cultured in maintenance media containing 10 μM ZD7288 continued to express the core pluripotency factors both on RNA and protein level. When the effect of treatment on differentiation was examined, application of the HCN blocker resulted in disparate outcomes for spontaneous FBS-driven differentiation, which was improved, and FBS-free directed differentiation to the neuroectoderm lineage, which was unaffected. Our findings underscore the importance of exploring the effect of small molecules on cell cycle progression and differentiation in a variety of culture contexts, and especially in serum-free culture conditions, which are of particular interest for further clinical development.

## Methods

### Cell culture

R1/E mouse embryonic stem cell line was purchased from ATCC (lot number: 3314644) and cultured as previously described (Andang et al. 2008b). In brief, cells were seeded at 7.5 × 10^3^ cells/ml in flasks for suspension culture or on laminin coated tissue culture plates for adherent culture (3 ml/well of 6-well plate, 0.5 ml/well of 24-well plate). Maintenance media consisted of DMEM/F12 (Gibco) supplemented with N2 media supplement (Gibco), HEPES (Gibco), beta-mercaptoethanol (Gibco), bFGF (R&D systems) and LIF (Millipore). Cells were grown at 37 °C, 20 % oxygen and split every 4 days using TrypLE Express (Gibco). HCN3 activity was inhibited by applying a specific small molecule inhibitor of the channel family, ZD7288 (Tocris), at a concentration of 10 μM in culture medium. Control and treated cells were counted and viability assessed at split using the NucleoCounter NC-3000 (Chemometec), and doubling time calculated using exponential regression in GraphPad Prism 6 (GraphPad Software Inc.). Single cells were fixed with 1 % PFA at RT for 15′ for flow cytometry analysis. For EdU incorporation, single cell suspension or adherent cells were incubated with EdU (Molecular Probes, product number: A10044) for 15′ prior to PFA fixation.

### Colony formation assay

To assay colony formation cells were plated on laminin coated 6-well/24-well tissue culture plates at 3 × 10^2^ cells/cm^2^. Colonies were allowed to grow with or without HCN3 inhibition for 5 days, at which point cells were fixed with 4 % PFA for 15′ at RT. For colony formation frequency, plates were stained for ALP activity (45′ incubation at RT in Whatman filtered solution of 0.01 g naphtol AS MX-PO_4_ (Sigma), 0.06 g red violet LB salt (Sigma), 400uL N,N-dimethylformamide in 50 ml 0.1 M Tris-HCl, pH8.3) and were imaged using the CellObserver (Zeiss) microscopy system, the number of colonies per well was counted manually and morphology scored. For marker expression, cells were grown on coverslips, stained, mounted on glass slides (VWR) using fluorescence mounting medium (Dako) and imaged on Zeiss LSM-710 confocal microscope.

### Differentiation studies

For differentiation studies cells were plated on laminin coated 24-well tissue culture plates at 1.5 × 10^4^ cells/cm^2^. FBS differentiation was carried out in DMEM/F12 containing HEPES, Glutamax (Gibco), and 15 % FBS (Gibco). Differentiation to the neural lineage was carried out in maintenance media supplemented with B27 (Gibco) and lacking bFGF and LIF, as previously described (Ying and Smith 2003). Media was changed every other day and RNA was collected 5 days after differentiation induction.

### Gene expression analysis

RNA from treated and untreated cells was collected and isolated using the Qiagen mini kit (Qiagen). 500 ng of total RNA was reversed transcribed (Applied Biosystems) and cDNA was used for qPCR reactions using SYBR green PCR master mix (Invitrogen) run on 7500 Fast Real-Time PCR system (Applied Biosystems). *Tbp* was used as the housekeeping gene reference. Primers sequences can be found in Additional file 1: Table S1.

### Antibody staining

Following PFA fixation cells were incubated for 1 h at RT with 1 % BSA and 0.1 % Tween-20 in PBS to permeabilize and block. Primary antibodies were diluted in the blocking solution and applied overnight at 4 °C. Cells were washed 2 times in PBS and species-matched secondary antibodies diluted in blocking solution was applied for 1 h at RT. Cells were incubated in 10 μM DAPI in PBS for 15′ at RT, washed twice and either mounted or analyzed using CyAN ADP flowcytometer (Beckman-Coulter). List of antibodies used can be found in Additional file 1: Table S2.

## Additional file

10.1186/s40064-016-1678-7 Primer sequences for qPCR and list of antibodies used.
