# Supplementary material for: ZD7288, a blocker of the HCN channel family, increases doubling time of mouse embryonic stem cells and modulates differentiation outcomes in a context-dependent manner
Source: Springerplus. 2016 Jan 16;5:41. doi: 10.1186/s40064-016-1678-7 (PMC4715829; doi:10.1186/s40064-016-1678-7)
Supplement: Supplementary file 1 — 10.1186/s40064-016-1678-7 Primer sequences for qPCR and list of antibodies used. [file 40064_2016_1678_MOESM1_ESM.docx]

Table S1. Primer sequences for qPCR.

| **Gene** | **Primer (fwd)** | **Primer (rev)** |
| --- | --- | --- |
| *Oct 4* | GCTCTCCCATGCATTCAAAC | TGTCTACCTCCCTTGCCTTG |
| *Sox2* | ATGGCCCAGCACTACCAGAG | CTTCTCCAGTTCGCAGTCCA |
| *Nanog* | TTTGGAAGCCACTAGGGAAAG | AAGCCCAGATGTTGCGTAAGT |
| *Rex1* | TTGATGGCTGCGAGAAGAG | GACTACCCAGCCTGAGGACA |
| *Gsc* | AAAGCCTCGCCGGAGAA | AGCTGTCCGAGTCCAAATCG |
| *T* | TCCTCCATGTGCTGAGACTTGT | TGCCACTTTGAGCCTAGAAGATC |
| *Fgf8* | CACAGAGATCGTGCTGGAGA | TGTACCAGCCCTCGTACTTG |
| *Sox17* | CCGAGATGGGTCTTCCCTAC | CGTCAAATGTCGGGGTAGTT |
| *Sox1* | CACAACTCGGAGATCAGCAA | CTCGGACATGACCTTCCACT |
| *Gata6* | GAACGTACCACCACCACCAT | CCATGTAGGGCGAGTAGGTC |
| *Bmp2* | GCTCCACAAACGAGAAAAGC | AGCAAGGGGAAAAGGACACT |
| *Hnf4* | AGTAACCTAGTCATGGCAAAGAAGATG | GGGCCTCACACCCTTTCTG |
| *Otx2* | AAATCAACTTGCCAGAATCCA | GGCCTCACTTTGTTCTGACC |
| *Tbp* | GGGGAGCTGTGATGTGAAGT | CCAGGAAATAATTCTGGCTCA |

Table S2. List of antibodies used.

| **Antigen** | **Species** | **Supplier** | **Product number** | **Dilution** |
| --- | --- | --- | --- | --- |
| SSEA1 | mouse | Abcam | ab16285 | 1:200 |
| OCT3/4 | rabbit | Santa Cruz | sc9081 | 1:500 |
| SOX2 | mouse | Abcam | ab79351 | 1:1000 |
| NANOG | rabbit | Abcam | ab80892 | 1:200 |
| KI67 | rabbit | ThermoFisher | RM-9106 | 1:1000 |
| gH2AX | rabbit | Abcam | ab2893 | 1:1000 |
| Rabbit IgG, Alexa488 | goat | ThermoFisher | A11034 | 1:1000 |
| Rabbit IgG, Alexa 488 | goat | ThermoFisher | A21428 | 1:1000 |
| Mouse IgG, Alexa 555 | goat | ThermoFisher | A21422 | 1:1000 |
